# A rapid-response soft end effector inspired by the hummingbird beak

**DOI:** 10.1098/rsif.2024.0148

**Published:** 2024-09-04

**Authors:** Jiajia Shen, Martin Garrad, Qicheng Zhang, Vico Chun Hei Wong, Alberto Pirrera, Rainer M. J. Groh

**Affiliations:** ^1^ Bristol Composites Institute (BCI), School of Civil, Aerospace and Design Engineering, University of Bristol, Bristol BS8 1TR, UK; ^2^ Exeter Technologies Group (ETG), Department of Engineering, University of Exeter, Exeter EX4 4PY, UK; ^3^ Department of Engineering Mathematics, University of Bristol, Bristol BS8 1TR, UK; ^4^ SoftLab, Bristol Robotics Laboratory, University of Bristol and University of the West of England, Bristol BS8 1TR, UK; ^5^ Faculty of Science and Engineering, Swansea University, Swansea SA1 8EN, UK

**Keywords:** functional morphology, snap-through instability, elastic tailoring, programmability, energy amplification, well-behaved nonlinear structures

## Abstract

Biology is a wellspring of inspiration in engineering design. This paper delves into the application of elastic instabilities—commonly used in biological systems to facilitate swift movement—as a power-amplification mechanism for soft robots. Specifically, inspired by the nonlinear mechanics of the hummingbird beak—and shedding further light on it—we design, build and test a novel, rapid-response, soft end effector. The hummingbird beak embodies the capacity for swift movement, achieving closure in less than 10ms . Previous work demonstrated that rapid movement is achieved through snap-through deformations, induced by muscular actuation of the beak’s root. Using nonlinear finite element simulations coupled with continuation algorithms, we unveil a representative portion of the equilibrium manifold of the beak-inspired structure. The exploration involves the application of a sequence of rotations as exerted by the hummingbird muscles. Specific emphasis is placed on pinpointing and tailoring the position along the manifold of the saddle-node bifurcation at which the onset of elastic instability triggers dynamic snap-through. We show the critical importance of the intermediate rotation input in the sequence, as it results in the accumulation of elastic energy that is then explosively released as kinetic energy upon snap-through. Informed by our numerical studies, we conduct experimental testing on a prototype end effector fabricated using a compliant material (thermoplastic polyurethane). The experimental results support the trends observed in the numerical simulations and demonstrate the effectiveness of the bio-inspired design. Specifically, we measure the energy transferred by the soft end effector to a pendulum, varying the input levels in the sequence of prescribed rotations. Additionally, we demonstrate a potential robotic application in scenarios demanding explosive action. From a mechanics perspective, our work sheds light on how pre-stress fields can enable swift movement in soft robotic systems with the potential to facilitate high input-to-output energy efficiency.

## 1. Introduction

**Figure 1.**
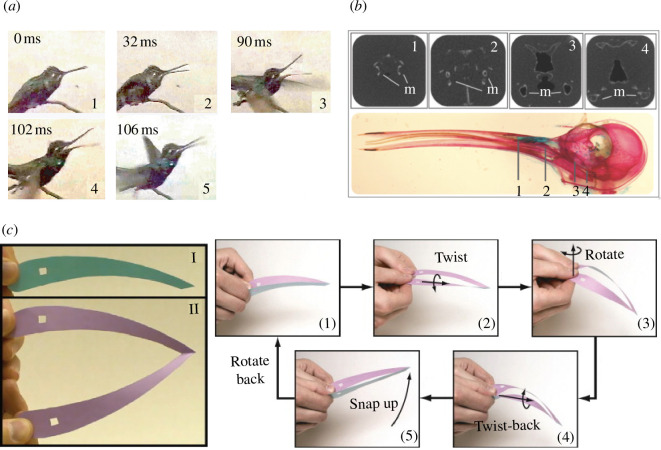
(*a*) The fast closure of the lower jaw of the hummingbird beak while capturing an insect. (1) Reference state; (2) the root of the beak is twisted; (3) the root of the beak is rotated at constant twist; (4) the root of the beak is twisted back at constant rotation; (5) the beak shuts via snap-buckling. (*b*) A lateral view of a cleared and stained hummingbird skull (red, bone; blue, cartilage) with cross-sections at four locations. The lower jaw of the beak comprises two branches, with high vertical bending stiffness but low horizontal bending stiffness. (*c*) Representative model of the hummingbird lower jaw based on a folded piece of paper. Square holes near the roots are present as a reference, but do not affect the model performance. (I) Side view (folded flat). (II) Top view (opened up). (1)–(5) Flexion and snap sequence produced by twisting and rotating the roots of the paper model. (1) Reference configuration. (2) The roots are twisted out. (3) The roots are rotated while twist remains unchanged. (4) The rotation is held fixed while the roots are partially twisted back. The paper model snaps between panels (4) and (5). (5) The roots remain rotated out. By rotating the roots back, the structure returns to the reference configuration. Photos (*a, b*) courtesy of Prof. Matthew L. Smith from Hope College, USA. Photo (*c*) is reproduced from Smith *et al*. [5] with the authors’ permission.

Explosive behaviours, such as jumping, striking, throwing and kicking, require high-power actuators to generate high accelerations [1]. Soft-bodied robots are limited in their performance of such tasks by the low power density of current artificial muscles [2]. Biological systems overcome the limited power density of their muscles by, for example, combining elastic elements with latching mechanisms that allow the slow build-up and rapid release of elastic energy [3,4]. Structural instabilities are another effective mechanism to enable rapid motion or shape adaptation [5–10]. Many of the systems exploiting instabilities to achieve rapid motion use simple and well-understood components, such as bistable beams or shells, and employ their canonical snap-through instabilities [11]. Accurate mathematical models have been developed to describe the nonlinear mechanics of these snap-through systems and, utilizing advanced materials [12] and actuation methods [13], to design rapid shape adaptation into engineering devices. Engineering structures that exploit buckling instabilities for functionality have been encapsulated under the rubric of ‘well-behaved nonlinear structures’ [14] or under the paradigm of *buckliphilia* [15]. Even though engineering systems based on bistable or multi-stable shells or post-buckled beams are coming to fruition [16], the mechanics governing many of their biological counterparts remains to be understood owing to their complexity in topology, material arrangement, applied loading/actuation and/or non-trivial bifurcation manifolds [17]. This lack of understanding limits their suitability for bio-inspired design. In this paper, we focus on the nonlinear mechanics of the rapidly closing beak of the hummingbird, which, as shown in figure 1*a*, shuts in merely 10 ms [5]—the proverbial ‘blink of an eye’.

**Figure 2.**
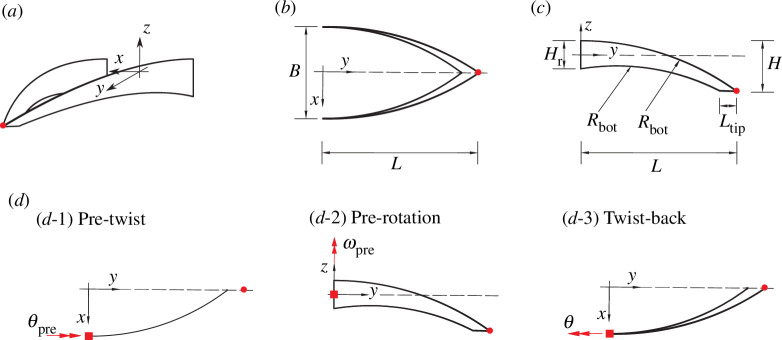
Geometry of the hummingbird beak model and the definition of the actuation inputs at the root section. (*a*) Three-dimensional view. Note the overall beak geometry is symmetric about the *y*–*z* plane. (*b*) Top view. We define the beak segment in the positive x -axis regime as the right branch and the beak segment in the negative x -axis regime as the left branch. (*c*) Side view of the right branch. The red dot at the tip of the beak is used in FE simulations and experimental tests as metric to quantify the deformation of the beak. (*d*) The actuation inputs at the root section. The double arrows represent the direction of the moment based on the right-hand rule.

**Figure 3.**
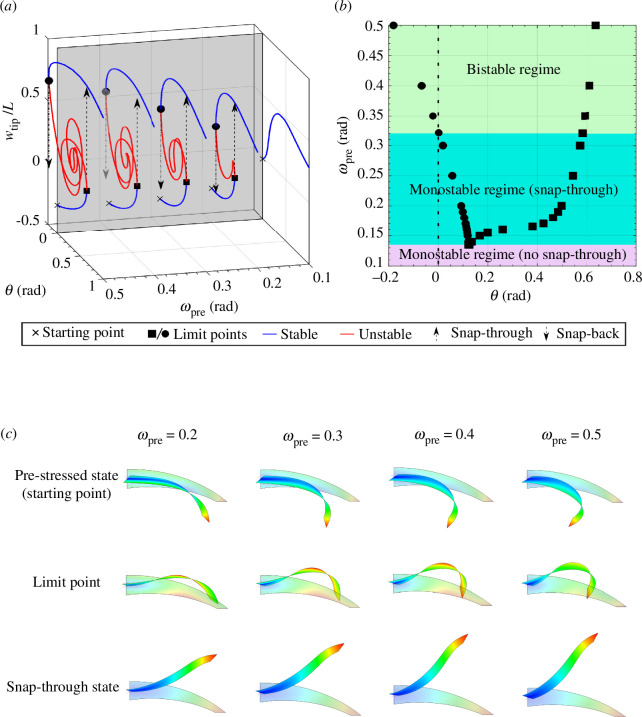
(*a*) The vertical displacement of the tip (normalized by beak length) versus the twist-back inputs θ at the root-section of the beak with pre-twist $\theta_{\mathrm{pre}}=2\;\mathrm{rad}$ and pre-rotations $\omega_{pre}=[0.1,0.2,0.3,0.4,0.5]\;rad$ . The blue and red curves represent stable and unstable equilibria and black dots represent limit points. We define the shape-shifting instabilties at the limit point squares and circles as ‘snap-through’ and ‘snap-back’ instabilities, respectively. Both twist and rotation inputs are in the global coordinate system. A grey reference plane with θ=0rad has been added to show the pre-stressed state. Equilibrium paths corresponding to $\omega_{\mathrm{pre}}=0.4$ and 0.5 rad traverse the reference plane. (*b*) The evolution of the limit points for different levels of pre-rotation. At each $\omega_{\mathrm{pre}}$ , the limit points on the right- and left-hand side of the plot correspond to snap-through and snap-back, respectively. The pair of limit points merge at a cusp catastrophe $\omega_{\mathrm{pre}}=0.133\;\mathrm{rad}$ , where snap-through vanishes. The snap-back limit point occurs at θ=0rad when $\omega_{\mathrm{pre}}=0.326\;\mathrm{rad}$ , and this level of pre-rotation is the minimum required for bistability. The beak-inspired structure with bespoke pre-stress state $\theta_{\mathrm{pre}}=2\;\mathrm{rad}$ can thus be classified into three characteristic zones as indicated by the colour coding. Note that the classification is made regarding the state with θ=0rad . The dashed line with θ=0rad corresponds to the grey reference plane in (*a*). (*c*) The deformation profile of the beak at selected states with different pre-rotation levels. The undeformed configuration is shown in translucent colours.

**Figure 4.**
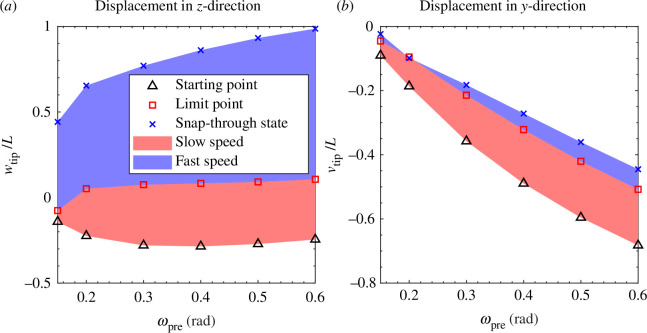
(*a*) Vertical ( z -direction) $w_{\mathrm{tip}}$ and (*b*) horizontal ( y -direction) $v_{\mathrm{tip}}$ displacement of the beak tip for four different pre-rotation levels. The red regime is the part of the twist-back procedure required to reach the snap-through instability and is determined by the speed of the applied rotational input. In contrast, the blue regime is characterized by the ensuing snap-through instability, and the resulting speed is mainly determined by the energy released.

**Figure 5.**
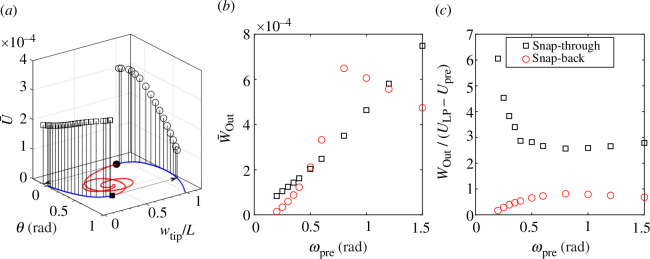
(*a*) The normalized strain energy ( $\bar{U}=U/(E\cdot V)$ ), where E is the Young’s modulus of the material and V is the volume of the beak-inspired structure, at stable equilibria for the beak with pre-twist $\theta_{\mathrm{pre}}=2\;\mathrm{rad}$ and pre-rotation $\omega_{\mathrm{pre}}=0.4\;\mathrm{rad}$ . Snap-through and snap-back are indicated using dashed arrows. The hollow squares denote strain energy levels before snap-through (up to the limit point (solid square)), and the hollow circles denote strain energy after snap-through. In particular, the hollow circle and square with the lowest U represent the restabilized equilibria after snap-through and snap-back, respectively. The difference between the strain energy at the limit points and the restabilized equilibrium denotes the energy released $W_{\mathrm{out}}$ upon snap-through or snap-back. For snap-through, the strain energy required to reach the limit point from the unactuated pre-stress state ( θ=0 ) is the energy input $W_{\mathrm{in}}$ ; for snap-back, the energy input $W_{\mathrm{in}}$ is the difference between the strain energy at the restabilized equilibrium (hollow circle with the lowest $\bar{U}$ ) and the limit point (solid circle). The dashed arrows represent the snap-through and snap-back events. (*b*) The normalized released strain energy ( ${\bar{W}}_{out}=W_{out}/(E\cdot V)$ ) during snap-through (black squares) and snap-back (red circles) at different pre-rotation levels $\omega_{\mathrm{pre}}$ . (*c*) Ratio of energy released from the snap-through/snap-back instability and the strain energy difference $\left(U_{\mathrm{LP}}-U_{\mathrm{pre}}\right)$ , which represents the energy input required to induce the snap-through or snap-back from the pre-stressed or snap-through restabilized states.

To the best of the authors’ knowledge, Smith *et al.* [5] were the first to describe the nonlinear mechanics of the swift closure of the hummingbird beak. In [5], the authors demonstrate that swift beak closure is achieved mainly through rapid movement of the lower jaw, which is depicted in figure 1*b*. They looked into the anatomical structure of the beak, focusing on the cross-sectional profile of the lower jaw, and identified that the structure is particularly compliant in the spanwise direction and stiffer in the orthogonal direction. Based on this observation, Smith *et al.* proposed a paper model that mirrors the mechanics of the hummingbird’s jaw, as shown in figure 1*c*. The complexity of the muscle action was simplified and reduced to three separate, sequential actuation steps, i.e. three rotations applied at the two roots of the beak: (i) a twisting motion about the axis of the beak, see figure 1*c*—(2); followed by (ii) an in-plane rotation, see figure 1*c*—(3); and finally (iii) a twist-back step that induces the dynamic snap-through instability, see figure 1*c*—(4) and (5). Based on insights from the paper model, Smith *et al.* [5] developed an illustrative mechanical model comprising rigid rods connected by hinges and torsional springs. Deriving the governing equations for the simplified model, its equilibrium manifold was determined using numerical continuation. This result revealed that the first two actuation steps introduce pre-stresses and stored elastic energy in the system, which is then released and converted into kinetic energy in the third step. In particular, the rotation introduced in the second step is crucial for creating a total potential energy surface with two wells, i.e. bistability. As is required for bistability, the two stable equilibria are separated by an energy barrier, which results in a saddle-node bifurcation in load-displacement space and, in turn, in fast snap-through dynamics. Smith *et al.* demonstrated the actuation and the snap-through instability of the beak phenomenologically, by means of simplified models. While these simple and elegant models can provide qualitative insight into the underlying mechanics of the hummingbird beak, their simplified nature limits their applicability for designing engineering systems that exploit the same mechanics. Hence, for the design of beak-inspired devices, a more representative numerical model is required. Interestingly, much effort has been placed on developing simpler shape-shifting systems, such as ‘hair clip’-inspired structures [18–20], which have many commonalities with the hummingbird beak but also reduced complexity in morphology and actuation. These systems operate under the general principle of ‘*stiffness tailoring*’ [12], whereby the nonlinear structural responses are tailored through bespoke pre-stress fields. This design philosophy has been widely used in laminated composite structures, exploiting differential thermal expansion between layers to induce multi-stability [14,21–23].

Our objective herein is to design a fast-response soft end effector, inspired by the snap-through mechanics of the hummingbird beak, which exploits the aforementioned concept of pre-stress enabled bistability. Both the morphology and input sequence applied to trigger snap through mirror the biomechanics of the hummingbird beak. To design the end effector, we develop a parametric finite element (FE) model that is used to tailor the bifurcation manifold that governs the snap-through event. Using the nonlinear solver in Abaqus CAE, we study the key actuation input affecting the response of the beak-like structure and its energy efficiency in terms of kinetic energy released upon snap-through as a ratio of the actuation energy supplied. A prototype soft-matter end effector is then manufactured and tested to validate the developed FE model and to demonstrate the programmable release of kinetic energy through elastic tailoring. As such, the present work provides direct guidance for designing fast-response soft end effectors that exploit snap-through mechanics. Furthermore, the insights provided by the FE models help to explain the biomechanics of hummingbird beaks, such as the greater energy efficiency of snap-through compared with snap-back of this particular morphology.

The rest of the paper is structured as follows. Section 2 provides details on the geometry of the beak model, the FE model development and the analysis procedure. In §3, parametric sensitivity studies are presented to: (i) identify the key actuation parameters governing the behaviour of the beak-inspired structure; and (ii) quantify its input-to-output energy efficiency. Section 4 presents a three-dimensional printed prototype end effector, made of thermoplastic polyurethane. The snap-through behaviour of the end effector is experimentally demonstrated and correlated against FE design predictions. Section 5 then presents two demonstrator examples of the developed end effector within a soft robotics context; first as a striking application and then as a catapult. We discuss the results in §6 and draw conclusions in §7.

## 2. Method

### 2.1. Geometry and actuation of the beak-inspired structure

Figure 2 presents the geometry of the hummingbird beak-inspired model as originally developed by Smith *et al.* [5]. The geometry is symmetric about the *y*–*z* plane. The model reflects the key geometric parameters and bending stiffness characteristics of the lower jaw of the hummingbird beak shown in figure 1*b*. The baseline geometry is described using the width, B , and height, $H_{r}$ , of the root section; the overall length, L ; total height, H ; and the length of the tip connection, $L_{\mathrm{tip}}$ ; with specific values shown in table 1. A parametric study was conducted to examine the effect of geometric scaling. It was found that the overall response of the beak is qualitatively unchanged when all dimensions are scaled by the same amount. Further details on scaling can be found in the electronic supplementary material, together with a .stl file of the model.

**Table 1.** Geometric parameters of the beak as defined in figure 2. All dimensions in mm.

Total height	H	32.3
Height of the root-section	$H_{r}$	18.2
Width at the root	B	58.6
Total length	L	100.0
Tip length	$L_{tip}$	11.1
Top arc radius	$R_{top}$	171.0
Bottom arc radius	$R_{bot}$	144.4
Thickness	t	3.2

The physical prototypes manufactured for testing are made of Stratasys FDM TPU (thermoplastic polyurethane) 92A. For the purpose of FE analyses, the material is assumed to be linearly elastic and isotropic, with Young’s modulus E and Poisson’s ratio ν of 15.3MPa [24] and 0.48 , respectively. While a neo-Hookean material model was also considered, the quantitative differences were negligible compared with using a St Venant–Kirchhoff material law.

As mentioned in §1, Smith *et al.* [5] identified an actuation sequence, with moments applied at the beak’s root-sections, to trigger snap-through. The sequence consists of three steps as shown in figure 2*d*:

Step 1: *Twist* the root-sections about the y -axis ( $\theta_{\mathrm{pre}}$ ).
Step 2: *Rotate* the root-sections about the z -axis ( $\omega_{\mathrm{pre}}$ ).
Step 3: *Twist-back* the root-sections about the y -axis ( θ ).

Note that, throughout the actuation sequence, all rotations are always defined about the global coordinate axes. In each step, only one rotation is applied while the others remain constant. With appropriate amount of twist and rotation in steps 1 and 2, the beak undergoes snap-through in the third twist-back step. Hence, from the viewpoint of elastic tailoring [12], the first two steps are required to introduce a pre-stress field that induces the desired nonlinear response upon twist-back. For this reason, we name the first two steps ‘pre-twist’ and ‘pre-rotation’, respectively. The state after pre-twist and pre-rotation is henceforth referred to as the initial state. The actuation step that induces the desired action, i.e*.* snap-through, is the *twist-back* step (variable θ ).

### 2.2. Finite element model development and solution technique

An FE model of the beak-inspired structure is developed within the commercial FE solver Abaqus CAE. To save computational effort, we adopt symmetry boundary conditions at the tip of the beak and only model one branch. Note that imposing this symmetry does not remove any symmetry breaking bifurcation (i.e. symmetry breaking bifurcations do not occur for the full model). The symmetric boundary conditions are set to reflect the compliance of the fold line at the tip of structure. That is to say, the fold line must remain in the y – z plane upon deformation but its geometry can change as the material along and around the fold deforms from its as-manufactured geometry. No additional stiffness was introduced at the fold line. Such modelling approach is widely used and validated for slender, thin-walled structures, where sections or even stiffeners are formed via folds [25].

A pre-processing script was developed to generate structured and uniform meshes with both shell (S4R) and solid (C3D8R) elements. A preliminary study on the accuracy of the FE models was conducted to identify the minimum required discretization fidelity. It was found that shell elements accurately depict the snap-through behaviour when compared with the higher fidelity solid elements. To save computational expense, shell element models were thus used for the ensuing phases of the study. Further details of the model can be found in the electronic supplementary material of this article. Mesh sensitivity studies were also conducted to determine a good element size to balance accuracy and computational effort, while guaranteeing converged results. For the baseline geometry with uniform rectangular cross-section, a mesh density of 30 shell elements in the z -direction yields converged results.

To apply rotations at the root-section, a reference point is placed at its centroid, see the red squares in figure 2*d*. This point is then linked to the other nodes along the same boundary via rigid body kinematic coupling. This constraint ensures that rotations applied at the reference point are uniformly transmitted to the entire root-section [26]. Referring to the coordinate system defined in figure 2, all translational degrees of freedom (d.f.s) of the reference point and its rotation about the x -axis are restrained. We adopt these ideal boundary conditions in the numerical analysis in §3 to unveil the governing mechanics of the beak-inspired structure. However, such boundary conditions are not easily implemented in an experimental set-up. In the experiments, we adopt a fixture to rigidly connect the root of the beak-inspired structure to the driving actuator. This fixture catches 15  mm of the beak-inspired structure at the root, constraining it to move almost rigidly. While changing the effective length of the beak in comparison with the idealized model of §3, this approach ensures that all the rotation input axes continue to pass through the root-section’s centroid, as shown in figure 2*d*. For accurate comparison with the experiments, the FE model is adapted accordingly, with the embedded parts constrained to move like rigid bodies with the root sections, where actuation inputs are applied as in the ideal model.

The response of the beak throughout the actuation sequence is traced using the multi-step analysis functionality in Abaqus. The effects of geometric nonlinearity are included by turning on the Nlgeom option in the solver. The rotational inputs are applied quasi-statically. The pre-twist and pre-rotation steps are solved using Abaqus’ Static General solver. The final twist-back step, where snap-through may occur, is solved using the static arc-length Riks solver [27] or the implicit dynamic solver with numerical damping to obtain quasi-static solutions. The static Riks solver can trace the complete unstable equilibrium path beyond the limit point (snap-through-inducing saddle-node bifurcation), while the implicit dynamic solver reproduces the snap-through behaviour observed in experimental tests. Note that in the implicit dynamic solver, we adopt the quasi-static setting with the keyword *Dynamic,application = QUASI-STATIC,initial = NO. This setting introduces significant numerical damping and removes the high-frequency dynamic component of the structural response, making it almost equivalent to a static analysis. Consequently, the velocities obtained from the numerical simulation do not directly reflect the actual velocities observed in the experiment. The quasi-static FE simulation focuses on revealing the beak-inspired structure’s nonlinear mechanics, while the dynamic behaviour induced by snap-through instability will be investigated using an experimental approach in §4.

## 3. Numerical results

In this section, we investigate how the pre-stress field introduced via pre-twist and pre-rotation affects the beak’s twist-back versus tip displacement equilibrium manifold, as well as the strain energy that is stored and then released upon snap-through. Previous work by Smith *et al*. [5] using a simplified analytical model has qualitatively demonstrated the equilibrium manifold of the beak structure under combined rotational actuation inputs (twist and rotation) and identified a folded equilibrium surface. Their findings unveil that the sequential pre-twist and pre-rotation inputs serve to move the beak from the lower to the upper surface of folded manifold. Notably, pre-rotation plays a more critical role in the energy release and fast response of the beak structure, compared with pre-twist. Our numerical study corroborates these observations. Moreover, we find that excessive pre-twist reduces the energy released. Therefore, our work focuses on the effects of pre-rotation on the beak’s behaviour. Details of a sensitivity study into pre-twist are provided in the electronic supplementary material.

### 3.1. Effect of the pre-actuation steps on the twist-back versus tip displacement equilibrium manifold

The beak-inspired structure remains statically stable (no negative eigenvalues in the tangent stiffness matrix as reported in the .msg file of Abaqus) throughout the pre-stressing steps (pre-twist and pre-rotation). We therefore focus on analysing the stability of the structure through the twist-back actuation step, which, conversely, features the dynamic snap-through that enables swift movement.

Figure 3*a* presents twist-back versus tip displacement equilibrium paths of the beak-inspired structure under twist-back actuation θ , for constant pre-twist $\theta_{\mathrm{pre}}=2\;\mathrm{rad}$ and pre-rotation inputs $\omega_{\mathrm{pre}}=\{0.1,0.2,0.3,0.4,0.5\}\;\mathrm{rad}$ . The choice of $\theta_{\mathrm{pre}}=2$ rad strikes a balance between efficiency and mitigating the effects of potential manufacturing imperfections.^1^ Note that θ represents the incremental twist angle from the initial state (after application pre-twist and pre-rotation), rather than an absolute angle. For each curve, the output displacement measure is taken to be the vertical ( z -axis) direction component of the displacement at the tip of the beak ( $w_{\mathrm{tip}}$ ).

For increasing $\omega_{\mathrm{pre}}$ , the beak transitions from an entirely stable response (e.g. $\omega_{\mathrm{pre}}=0.1\;\mathrm{rad}$ ) to increasingly complex nonlinear and hysteretic behaviour. Pairs of limit points appear along the equilibrium paths, the number of which increases for increasing $\omega_{\mathrm{pre}}$ . Each additional pair of limit points corresponds to an additional ‘loop’ in the unstable portion of the equilibrium path. The deformation profile of these unstable states corresponds to highly undulating bending modes along the beak, resembling the behaviour observed in snap-through arches under transverse loading [28,29]. Note that while these additional pairs of limit points were not observed in the simplified bar-spring model by Smith *et al.* [5], they do not lead to additional stable equilibria and therefore have little effect on the observed performance of the beak-inspired structure apart from corresponding to greater release of strain energy upon the loss of stability. As it is the limit points where exchange of stability occurs (stable to unstable and *vice versa*) that are important for the onset of dynamic snap-through, only these limit points are highlighted in figure 3*a*. To visualize the pre-stressed states, a grey reference plane at θ=0 has been added. Notably, the equilibrium paths corresponding to $\omega_{\mathrm{pre}}=0.4$ and 0.5 (rad) intersect the reference plane, indicating that the pre-stressed states for these two cases exhibit bistability.

Figure 3*b* presents the evolution of this pair of limit points for varying pre-rotation, $\omega_{\mathrm{pre}}$ . The limit points on the right- and left-hand side of figure 3*b* represent the beak-inspired structure exchanging stability, i.e. changing between stable and unstable. Here, we define the shape-shifting instabilities at the right-hand side (solid squares) and left-hand side (solid circles) limit points as ‘snap-through’ and ‘snap-back’ instabilities, respectively. The twist-back input, θ , required to traverse the first limit point and trigger the snap-through instability increases with $\omega_{\mathrm{pre}}$ . The twist-back value at the second limit point, which corresponds to a snap-back event as θ is decreased after snap-through, falls below zero when $\omega_{\mathrm{pre}}=0.326\;\mathrm{rad}$ . Hence, for pre-rotation $\omega_{\mathrm{pre}}>0.326\;\mathrm{rad}$ , the pre-stressed beak-inspired structure (with $\theta_{pre}=2\;rad$ ) is bistable, i.e. there are two stable configurations for θ=0rad . This means that if the bio-inspired soft end effector is to be designed to operate in a reversible manner using only mono-directional root actuation (i.e. θ>0 ), then the applied pre-rotation has to be smaller than 0.326rad . If pre-rotation is greater than this threshold, the actuation input driving θ needs to be capable of applying negative rotation.

The pair of limit points merge at $\omega_{\mathrm{pre}}=0.133\;\mathrm{rad}$ , a feature known as a ‘cusp’ catastrophe. When the pre-rotation level is below this cusp critical point, the beak does not exhibit snap-through in the twist-back actuation step. The cusp also describes the minimum pre-twist $\theta_{\mathrm{pre}}$ input required to allow snap-through, which for the present system is given by $\theta_{\mathrm{pre}}>1.88\;\mathrm{rad}$ . A detailed sensitivity study of $\theta_{\mathrm{pre}}$ can be found in the electronic supplementary material. For figure 3*b*, where $\theta_{\mathrm{pre}}=2\;\mathrm{rad}$ , there are three characteristic zones corresponding to qualitatively different responses: monostable without snap-through, monostable with snap-through, and bistable with snap-through. The performance of the beak-inspired structure can thus be tailored, or programmed, by adjusting $\omega_{\mathrm{pre}}$ . Figure 3*c* shows the deformation of the beak-inspired structure at selected states along the equilibrium manifold. With increasing pre-rotation, the beak is more deformed, and therefore more strain energy is stored through elastic deformation. Strain energy stored in the beak is released and transformed into kinetic energy that leads to the swift shape-shifting response of the beak. This is in accordance with the behaviour observed in the simplified analytical model by Smith *et al.* [5].

Figure 4 shows the tip displacement of the beak-inspired structure in both the vertical direction ( z ) and horizontal direction ( y ) at three characteristic stages: (*i*) the starting point (pre-stressed, initial state), (ii) the snap-through limit point, and (iii) the post snap-through state, for different pre-rotation levels. Generally speaking, as pre-rotation increases, the overall beak tip deformation increases in amplitude. Notably, the regime between $\omega_{pre}=0.15\;$ to 0.2rad , i.e. in proximity of the cusp, exhibits significant variation, which coincides with the dramatic shift observed in the limit points (square symbols) of figure 3*b*. The displacement dynamics can be divided into two stages: (i) the slower speed (kinematic) stage from the starting point to the snap-through limit point, where the speed is determined by the actuation input at the beak root, and (ii) the faster speed (dynamic) stage from the limit point to the post snap-through state, where strain energy is released and transformed into kinetic energy. The kinematic displacement amplitude at the beak tip $w_{\mathrm{tip}}$ in the first stage is almost independent of the pre-rotation, but the dynamic displacement in the second stage increases with increasing pre-rotation. Assuming that the speed of the rotation-control actuation motor is fixed, the time required for the first (kinematic) stage is predominantly influenced by the twist-back angle required to reach the snap-through limit point. The snap-through limit points on the right-hand side of figure 3*b* show an increase in twist-back required to reach the snap-through point as the pre-rotation angles increase. Notably, within the pre-rotation regime of $\omega_{pre}=[0.15,0.17]\;rad$ , the snap-through limit points in figure 3*b* show a steep increase in twist-back ( θ ) required to reach the snap-through point as pre-rotation angles $\omega_{\mathrm{pre}}$ increase.

These observations have implications for engineering applications and also provide some insight into potential evolutionary drivers of the hummingbird beak function. If our goal is to use the beak-inspired structure for rapid shape shifting, we need to consider that larger pre-rotations imply a double time penalty in that it takes longer to set the higher pre-rotation and longer to reach the snap-through point while applying the twist-back. Similar arguments are true for the natural setting of the hummingbird beak. The primary function of the hummingbird beak is to capture insects. If the time taken to pre-load the beak in the first stage is too large, insects may detect movements and escape.

Another consideration for potential engineering applications is the effective length of the beak-inspired structure, as shown in figure 4*b*. Although the larger strain energy stored in longer beaks allows for a speedy response, this comes at the cost of a decreased effective length owing to a distorted lengthwise geometry from higher order bending modes. For example, for $\omega_{\mathrm{pre}}=0.4\;\mathrm{rad}$ , the effective length of the beak after the pre-stressing step is half of the original length. This reduced effective length may not be suitable for certain applications and an optimized pre-rotation input may need to be determined by a compromise between these factors.

### 3.2. Energy transformation and efficiency

To better understand the working mechanism of the beak, we compare the energy storage and transformation efficiency of the different actuation stages. Before snap-through occurs, all of the work done by the externally applied actuation inputs is stored as strain energy in the structure. When snap-through occurs, the work done by the external, rotation-controlled actuation is zero as the rotation is fixed at a value just above the snap-through state. The strain energy released upon snap-through is thus equal to the difference between the strain energy at the limit point and the equilibrium state into which the structure restabilizes (at the same rotation). As Abaqus outputs the strain energy at each converged equilibrium state, this difference is readily computed.

Figure 5*a* presents the normalized strain energy, $\bar{U}=U/(E\cdot V)$ with U being the strain energy and V being the beak volume, along the stable segments of the equilibrium manifold for the beak-inspired structure with pre-twist $\theta_{\mathrm{pre}}=2\;\mathrm{rad}$ and pre-rotation $\omega_{\mathrm{pre}}=0.4\;\mathrm{rad}$ . The normalization factor is chosen based on a scaling sensitivity study. This ensures that the results are independent of both material properties and geometric scale. For both segments, the strain energy of the beak-inspired structure from the restabilized equilibrium states (snapped from the other stable segment) up to the snap-through or snap-back limit points is provided. For a direct comparison of energy transformation efficiency between snap-through and snap-back mechanisms, we exclude the energy required to set the pre-stressed state. A detailed discussion on overall energy efficiency, including the contribution from pre-stressing procedures, is provided later in this section. As expected, the strain energy at the snap-through limit point is higher than that at the initial pre-stressed state ( θ=0 ), as shown by the hollow squares. The increase in strain energy between these two states is the energy input required to trigger snap-through, which is denoted by $\Delta U=U_{\mathrm{LP}}-U_{\mathrm{pre}}$ . The strain energy at the snapped-through state (see the lowest hollow circle on the $\bar{U}$ -axis) is lower than at the snap-through limit point (highest hollow square on the $\bar{U}$ -axis) and the energy difference is the energy released upon snap-through, which is denoted as $W_{\mathrm{out}}$ . To amplify energy output, we aim for a large $W_{\mathrm{out}}$ as well as large energy efficiency $W_{\mathrm{out}}/\Delta U$ , i.e. also low ΔU . Figure 5*b*,*c* shows ${\bar{W}}_{\mathrm{out}}$ and $W_{\mathrm{out}}/\Delta U$ of the beak-inspired structure for different pre-rotation levels $\omega_{\mathrm{pre}}$ (see hollow squares). Generally, the energy released increases with $\omega_{\mathrm{pre}}$ . The efficiency initially decreases sharply and then stabilizes around 2.5. As a result, large energy output (requiring large $\omega_{\mathrm{pre}}$ ) is not equivalent to the most efficient operation (requiring low $\omega_{\mathrm{pre}}$ ). Figure 5*c* also shows energy ratios greater than 1 ( $W_{\mathrm{out}}/\Delta U>1$ ) because ΔU only accounts for the twist-back energy required to reach the snap-through limit point and does not include the pre-twist and pre-rotation steps, since these pre-stress energy values would be locked-in during the manufacturing and assembly of the device [30].

Figure 5*b*,*c* also presents the energy released and energy efficiency of the snap-back procedure (red hollow circles), i.e. to return to the initial pre-stressed state. Regarding the computation of ΔU , the restabilized equilibrium from the snap-through instability is selected as the starting pre-stressed state. The energy input to trigger snap-back is generally higher and the energy efficiency lower than for the snap-through sequence. In contrast to the snap-through scenario where released energy monotonically increases with increasing pre-rotation $\omega_{\mathrm{pre}}$ , the snap-back instability exhibits a non-monotonic trend. The released energy initially rises with $\omega_{\mathrm{pre}}$ but then decreases. This can be attributed to excessive structural deformation beyond a certain pre-rotation threshold. Specifically, the tip potentially traverses past the root. This behaviour is of little practical significance for the intended function of the beak-inspired structure.

**Figure 6.**
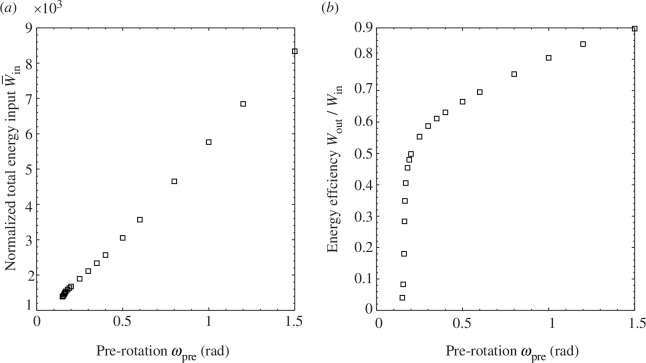
(*a*) Normalized energy input ( ${\bar{W}}_{\mathrm{in}}=W_{\mathrm{in}}/(E\cdot V)$ ) from the stress-free state to trigger the snap-through instability for the beak-inspired structure with different pre-rotation levels. Note that the pre-twist $\theta_{pre}=2\;rad$ in all cases. (*b*) The energy efficiency: the ratio of the energy released from the snap-through instability and the energy input $W_{\mathrm{in}}$ .

This observation also provides further insight into the biomechanics of the hummingbird beak. Based on the above energy efficiencies, we would expect the hummingbird to use snap-through, rather than snap-back, to catch its prey, and this is precisely how the hummingbird prey-catching mechanism functions. Based on these observations, we therefore focus on exploiting the snap-through instability in our biomimetic demonstrators. In addition, we find that restoring the beak-inspired structure to its initial pre-stress state by releasing all actuation inputs at the root-sections and reintroducing the pre-stress field from a stress-free state is more energetically efficient than returning the system via the snap-back instability. This method again mirrors the actuation sequence used by hummingbirds [5]. Figure 6 presents the energy input from the stress-free state and energy transformation efficiency for the snap-through instability for different pre-rotation levels. We observe a general trend of increasing efficiency with increasing pre-rotation. A particularly sharp rise in efficiency occurs from the cusp point up to $\omega_{pre}=0.2\;rad$ , which correlates with the sharp change in the fold line presented in figure 3*b*. Above $\omega_{pre}=0.4\;rad$ , the efficiency exhibits a linear increase. Notably, at $\omega_{pre}=1.5\;rad$ , the energy transformation efficiency approaches 90%. This efficiency trend is opposite to that in figure 5*c*. This implies that a smaller proportion of the total input energy is required to reach the pre-stressed state, while a larger proportion is needed to actuate the beak from the pre-stressed state to the snap-through limit point. This aligns with the significant increase of the twist-back required to trigger the snap-through instability presented in figure 3*b*.

## 4. Experimental validation

To validate the numerical simulations and study the dynamic behaviour of the beak-inspired structure, we manufactured a set of prototypes using three-dimensional printing and designed an actuation system to apply the sequential rotations at both roots of the structure. The beak-inspired structure was manufactured with FDM TPU (Shore A hardness 92A) using fused deposition modelling (FDM) three-dimensional printing on a Stratasys Fortus 450mc printer. The pre-processing script that generates the input file for the FE model in Abaqus is modified to generate a stereolithography (.stl) file for three-dimensional printing. This unified pre-processing script ensures that the printed beak has the same geometric properties as the FE model. The actuation system and details of the manufacturing process are described in the electronic supplementary material.

**Figure 7.**
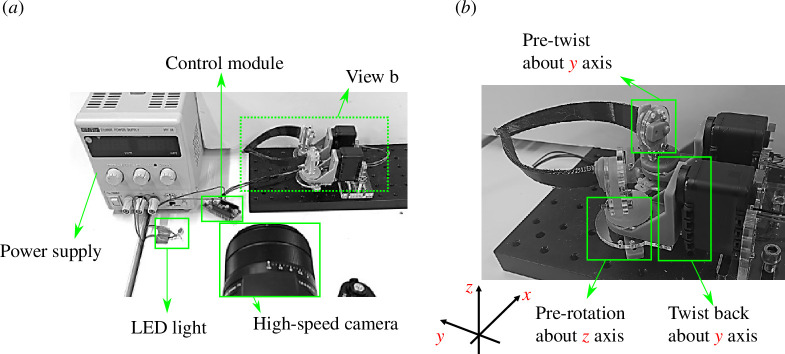
Experimental set-up: (*a*) Overview of the experimental set-up. Test rig connected with control module and power supply. LED light is used to provide signal for the data log synchronization. High-speed camera system is used to track the motion of the beak. (*b*) Actuation and supporting system to apply the pre-twist, pre-rotation and twist-back on the beak-inspired structure.

Figure 7 presents the experimental set-up for mechanical testing of the beak-inspired structure. As the pre-twist and pre-rotation inputs introduce the pre-stress field, these two actuations are applied by adjusting the gears manually and then locking them in place using bolts and pre-cut holes in the testing rig. The final twist-back step, through which snap-through is triggered, is actuated through two synchronized servo motors (Dynamixel AX12-A, Robotis, South Korea). The two roots of the beak are glued into the fixtures of the actuator systems. The depth of the fixture is 15 mm. The tightness between the fixture and the beak ensures that they are rigidly connected throughout the entire actuation process. The fixture set-up makes the boundary conditions of the test sample different from the ideal case as shown in figure 2 and discussed in §3. The axes of the pre-twist, pre-rotation and twist-back are aligned to ensure that they cross at the centroids of the root-sections of the beak-inspired structure.

In the measurement system, we use a high-speed camera (Sony Cyber-Shot DSC-RX10) to record the deformation profiles of the beak from a side view ( y – z plane) at 1000 frames per second. In particular, the displacement of the tip of the beak (labelled with a red dot for visualization purposes) as well as the average speed throughout snap-through is determined through an in-house image processing script in Python. Servo motor positions are recorded at 500Hz and an LED is placed in the frame of the high-speed camera and switched off at the onset of the twist-back step in order to synchronize the two data acquisition systems.

**Figure 8.**
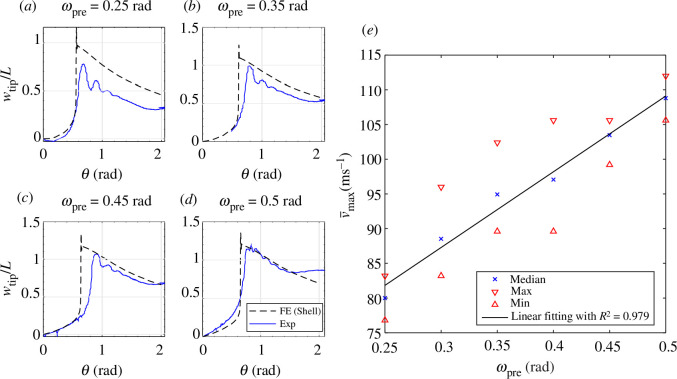
(*a–d*) Comparison between experimental and simulated response of the beak-tip vertical displacement versus twist-back actuation for different levels of pre-rotation $\omega_{\mathrm{pre}}$ . To ensure repeatability, three tests were conducted for each case, exhibiting minimal variation between measurements. For clarity, only a single representative curve is presented. Videos of these tests can be seen in the electronic supplementary material. Note that the boundary conditions of the FE model are different from that in the ideal case shown in figure 3a to reflect the actual boundary conditions in the mechanical testing set-up. (*e*) The peak velocity of the beak-inspired structure during snap-through for different pre-rotation inputs $\omega_{\mathrm{pre}}$ . Note that the velocity is normalized with the respect to the length of the beak L , i.e. ${\bar{v}}_{\max}=v/L$ . For each pre-rotation, three tests were conducted. A linear function is fitted to describe the median velocity versus the pre-rotation with $R^{2}=0.979$ .

In the numerical simulations, we identified that the pre-rotation input, $\omega_{\mathrm{pre}}$ , is crucial for swift movement. Therefore, the response of the beak with different $\omega_{\mathrm{pre}}$ levels is tested. Figure 8*a*–*d* presents the experimental equilibrium paths with $\omega_{\mathrm{pre}}$ equal to 0.25 , 0.35 , 0.45 and 0.5rad , alongside the FE simulation results using an implicit dynamic solver. Note that for each case, three repeated tests were conducted and minimal variation between repeated measurements was observed. For clarity, a single representative curve from the repetitions is presented. The experimental results show good correlation with the FE simulations, both before and after snap-through, for different levels of pre-rotation. We observe some oscillations in the experimental results upon snap-through corresponding to damped vibrations around the snapped-through equilibrium state. The observed oscillations probably stem from the steady twist-back actuation speed (set as 8.60 rad s^−1^). The extent of the oscillations could potentially be decreased by employing a reduced twist-back actuation speed during the experiment to minimize inertial effects. The largest difference between the FE results and the experiments is in the location of the snap-through point. Owing to storage memory limitations of the high-speed camera used in this study and the fact that any application of the beak-inspired structure as an end effector would require rapid actuation, we selected a fast actuation speed in the experiments. As a result, the quasi-static assumption made in the FE models is not met in the experiments and the dynamic experiments overshoot the quasi-static snap-through point owing to inertial effects. In the electronic supplementary material, we show that with decreasing experimental actuation speed the snap-through point in the experiments also correlates well with the FE simulations. The quasi-static equilibrium curves nevertheless serve as useful backbones to the dynamic experiments and the portion of the experimental data acquired before and after the snap-through point, which are less sensitive to actuation speed, correlate closely with the FE simulation and are thus sufficient to validate the FE model.

Generally speaking, the greater the pre-rotation $\omega_{\mathrm{pre}}$ the better the correlation between experiments and simulation. This is to be expected because reducing the pre-rotation places the system closer to the cusp where snap-through vanishes. The cusp is a codimension-2 bifurcation around which the system is especially sensitive to imperfections in the experimental set-up or the manufactured dimensions of the beak.

Figure 8*e* presents the vertical speed at the tip of the beak with different levels of pre-rotation $\omega_{\mathrm{pre}}$ . The peak velocity of the beak tip increases with increasing pre-rotation. The relationship between the median peak velocity and the pre-rotation in the regime presented can be approximated by a linear function to good accuracy ( $R^{2}=0.979$ ).

## 5. Demonstrations of soft robotics applications

We demonstrate the potential applications of the beak-inspired structure in two tasks where rapid energy release is of benefit: striking and throwing an object. It is important to acknowledge that in these scenarios, not all of the energy released during the snap-through instability translates directly into the desired functional output. Therefore, the data presented here serves as a qualitative demonstration of the mechanics explored in the preceding sections, focusing on velocity transfer at the beak tip.

### 5.1. Characterization of beak-inspired striking performance

**Figure 9.**
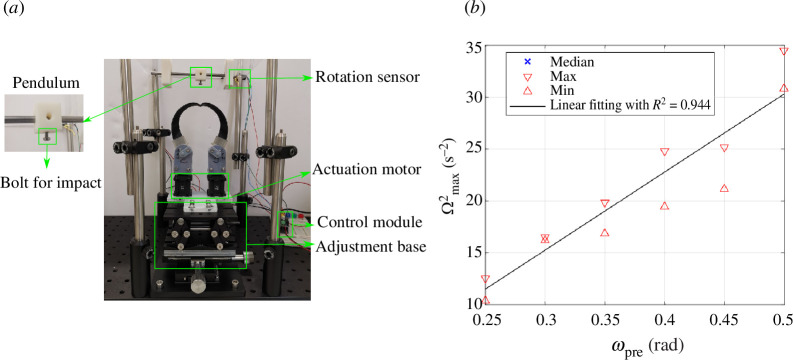
(*a*) Pendulum test set-up. The pendulum and bolt for impact are shown in a detailed view. A Hall effect angular sensor measures the movement of the pendulum. Two servo motors are used to apply rotational inputs. A multi-axis adjustment stage is used to adjust the position of the beak so that the beak can impact the pendulum at the lowest point that intersects with the pendulum. (*b*) The peak angular velocity squared of the pendulum after being hit by the beak tip for different levels of pre-rotation. A linear function is fitted to describe the median velocity squared versus the pre-rotation with $R^{2}=0.944$ .

To demonstrate the striking application, we show the beak hitting a pendulum. The test set-up is shown in figure 9*a*. This set-up allows us to measure the energy transferred by the beak to the pendulum by measuring the peak velocity of the suspended mass. The beak-inspired structure and the actuation system are the same as in the mechanical test of §4, but rotated by 90° to enable an impact normal to the pendulum’s resting position. The pendulum is rigidly connected to a smooth steel rod (6 mm diameter), with the rod connected to the test frame via two ball bearings. The mass of the rod is 25.21 g. The mass of the pendulum is 6.18 g and the length of the pendulum is 32 mm. A Hall effect angle sensor (Melexis mlx90363, Digikey, UK) is installed at the end of the smooth bar to measure its rotation and thereby the rotation of the connected pendulum as a result of the impact of the beak. As shown in §3, the deformed profile of the beak varies with pre-rotation input. The beak and its actuation system are embedded on an adjustable stage so that the pendulum can be aligned with the tip of the beak in its post snap-through equilibrium position. This position is typically below the path taken by the beak during snap-through, and so the beak is raised in steps of 2 mm until a reliable impact is achieved. We emphasize that this point may not correspond to the location of the peak tip velocity (and thus energy release), but is used here because it can be consistently and reliably determined. We also believe that this scenario is closest to how the beak may be used in a practical application, avoiding the need for a cumbersome alignment process after each adjustment of the beak pre-rotation.

For the beak-inspired structure we used herein, we found that the peak velocity occurs approximately when the first maximum horizontal displacement is reached. Further details can be found in the electronic supplementary material. Figure 9*b* presents the peak squared angular velocity of the pendulum for different levels of pre-rotation. With increasing pre-rotation, the peak velocity of the pendulum increases. The relationship of peak velocity squared to pre-rotation level can be approximately described by a linear relationship ( $R^{2}=0.944$ ), allowing for facile control of energy transfer during striking scenarios. The observed linear relationship between peak squared angular velocity and pre-rotation in figure 9*b* is reminiscent of the trend observed for peak tip velocity and pre-twist in figure 8*b*. However, it is crucial to recognize that these relationships are not directly proportional. Two key factors contribute to this distinction. First, the impact angle of the beak tip on the pendulum varies across different pre-rotation levels owing to the structures’ distinct deformation profiles at impact. Second, the efficiency of energy transfer during the impact also differs between the different cases.

Table 2 presents the maximum kinetic energy achieved by the entire pendulum system (including the pendulum and the rotating bar) at various pre-rotation levels. The comparison with the total strain energy released during the snap-through instability, which is computed using the quasi-static nonlinear finite element analysis, is also presented. The ratio of the pendulum’s maximum kinetic energy to the total released energy, is around 2%. This low value is understandable considering that only the beak tip impacts the pendulum, and the beak-inspired structure continues its forward motion after the impact. This low energy transformation efficiency suggests that the beak-inspired structure is ideally suited for applications where rapid shape-shifting is the primary function, such as in grippers [31]. Alternatively, the entire structure’s motion can be leveraged for tasks where overall body movement is the output, like the fin of a swimming robot [32].

**Table 2.** Energy transferred to the pendulum with varying pre-rotation levels and comparison of the energy released from the snap-through instability computed using the quasi-static nonlinear finite element analysis. The maximum kinetic energy is computed based on the median peak angular velocity presented in figure 9*b*. Note that pre-twist $\theta_{pre}=2\;rad$ in all cases.

pre-rotation $\omega_{pre}$ (rad)	maximum kinetic energy of pendulum $E_{k,p}$ (mJ)	$E_{k,p}/W_{out,FE}$
0.25	36.24	1.75%
0.3	52.44	2.14%
0.35	60.21	2.14%
0.4	72.62	2.27%
0.45	75.69	2.10%
0.5	104.12	2.60%

### 5.2. Demonstration of a beak-inspired catapult

**Figure 10.**
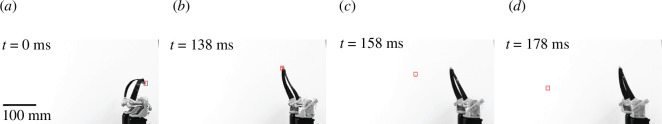
Snapshots of the ball thrower: (*a*) Pre-stressed state; (*b*) state before ball detached with the beak tip; (*c*) 20 ms after detachment; and (*d*) 40 ms after detachment. For easy visualization, the ball location is highlighted using a red box. Note that the pre-rotation in the beak-inspired structure is 0.45rad . The weight of the ball is 0.62 g. A movie of the demo ball thrower can be found in the video folder of the electronic supplementary material.

The beak-inspired structure can also be used for throwing or launching applications by modifying the beak into an elastic catapult. Owing to the complex shape of the beak tip, we applied a small amount of double-sided tape to the beak tip to hold a projectile. This provided sufficient adhesive force to hold a projectile (mass of 0.62 g) on the tip of the beak prior to launch. Upon applying the twist-back step beyond the stable limit point, the rapid acceleration of the beak tip and the dramatic change of the adhesive area owing to the shape change of the beak meant the inertial forces of the projectile were able to overcome this adhesive force, allowing launching of the projectile. Figure 10 presents the trajectory of the ball projectile using sequential photos, where the pre-twist and pre-rotation in the beak are 2 rad and 0.45 rad, respectively. The projectile is launched at approximately t=138ms , see figure 10*b*. The twist-back angle at the root at this instant is close to the value of the measured peak velocity in the mechanical testing in §4, in order to maximize the launch velocity of the projectile. A full video featuring application of the beak-inspired end effector in this throwing scenario can be found in the video folder of the electronic supplementary material. Based on these videos, we estimate the peak velocity of the projectile to be 5.5 m s^−1^ corresponding to a kinetic energy of 9.4 mJ. This is about half the peak tip velocity (around 10.37 m s^−1^) observed in the mechanical testing with no projectile (refer to figure 8*b*). Compared with the pendulum case, the energy transfer efficiency for projectile launching is even lower. This can be attributed to energy dissipation owing to the adhesive force between the double-sided tape and the projectile, which the beak-inspired structure needs to overcome for launch. At lower pre-rotation levels, the projectile was not launched at all, probably owing to insufficient energy to overcome the adhesive force and achieve projectile detachment.

## 6. Discussion

Previous research has used latch systems [33,34] to store strain energy and achieve explosive energy output, e.g. for jumping robots. The present beak-inspired structure achieves high energy output without a latch system, thereby simplifying the design. Rather than using a latch-and-release mechanism to store strain energy that is then rapidly converted to kinetic energy, the beak-inspired structure achieves the same functionality through bistability, i.e. an elastically tailored energy barrier. The explosive energy output, i.e. snap-through behaviour, only occurs when the energy barrier is overcome by a desired and large actuation input. Both the energy output and the magnitude of the energy barrier can be tailored through the pre-stress field.

The structural topology, including the overall shape and cross-section profile of the structure, also significantly affects the snap-through response. For instance, computed tomography (CT) scans of hummingbird beaks [35] reveal that the cross-sectional profile along the length of the beak resembles a C-section with mass concentrated at the top and bottom of the section. This morphology is fundamentally different from the rectangular cross-section adopted for our studies herein. Although the beak’s geometrical profile may not solely serve the purpose of rapid shape-shifting, further investigation into the effects of geometric properties is necessary that may then influence the design of even more efficient rapid response end effectors.

The finite element model in this paper focuses mainly on recovering quasi-static responses and the energy released from the snap-through instability. While this approach provides valuable insights, it does not fully capture the dynamic behaviour of the beak. To address this limitation, future work will incorporate the beak’s inertial and damping characteristics in an explicit dynamic analysis to provide a more comprehensive understanding of the beak’s performance during the rapid snap-through process.

Here, both pre-stressing and shape-shifting are achieved through actuation inputs at the root of the beak-inspired structure. A potentially simpler solution is to introduce an equivalent pre-stress field during manufacturing [12], for example, by using fibre-reinforced composite materials. These materials have orthotropic properties and by varying properties in-plane or through the thickness, tailored pre-stress distributions can be induced during post-cure cooling [23]. This approach could simplify the actuation system and enable more efficient and precise control of the beak-inspired structure.

In our present work, the beak-inspired end effector is actuated by rotations applied at the root. Another means of actuation may be to use embedded actuation techniques [30] using active materials [36], which could potentially make the system stand alone without requiring external actuation.

## 7. Conclusion

Through detailed nonlinear finite element simulations and experimental testing, we have confirmed the hypothesis of Smith *et al.* [5], explored therein using a simplified model of rigid links and hinges, that the response of a hummingbird beak-inspired structure can be tailored using a pre-stress field induced by modulating the pre-twist and pre-rotation at the root sections of the structure. In addition, our models and experiments reveal that the beak-inspired structure can store and quickly release elastic energy with a single actuation input. This mechanism can potentially amplify the power of soft, artificial muscles and improve the ability of soft-bodied robots and end effectors to accomplish explosive behaviours.

We identified that the both pre-twist and pre-rotation steps are important for triggering of snap-through behaviour, and the pre-rotation step is crucial for tailoring the snap-through behaviour. Under a certain threshold value, no snap-through is possible, i.e. the beak-inspired structure is monotonically stable under actuation. Equally, above a second threshold the structure is bistable in both unactuated and actuated snap-through states. The dynamic energy released through the instability increases as the pre-rotation is increased.

Based on the numerical results, we designed and manufactured a beak-inspired end effector for soft robotic applications with an external actuation system and verified the fast response experimentally. The dynamic response time from original to snapped-through state can be in the magnitude of 10 ms. This provides a potential solution for fast-response grippers and actuation systems in robotic systems.

In addition, our simulation and experimental findings provide potential explanations for certain aspects of the hummingbird beak’s biomechanics. We have shown that this particular structure has greater energy efficiency when snapping through (positive twisting) than when snapping back, which agrees with the operation of hummingbird beaks. Secondly, the most efficient means of repeatedly actuating the beak-inspired end effector is to restore the structure to its initial pre-stress state by releasing all actuation inputs, which again agrees with biomechanical observations of the hummingbird beak.

Future research will focus on the development of buckling-driven adaptive systems with simpler geometry, easier manufacturability and less complex actuation inputs based on stiffness tailoring.

## Data Availability

Data (including the videos about the experiment) are available from Dryad [37]. Supplementary material is available online [38].

## Footnotes

^1^
The beak-inspired structure is sensitive to imperfections if the pre-stressed state is in proximity of the cusp, potentially precluding the snap-through instability.

## References

[1] Hawkes EW , Xiao C , Peloquin RA , Keeley C , Begley MR , Pope MT , Niemeyer G . 2022 Engineered jumpers overcome biological limits via work multiplication. Nature **604** , 657–661. (10.1038/s41586-022-04606-3) 35478234

[2] El-Atab N , Mishra RB , Al-Modaf F , Joharji L , Alsharif AA , Alamoudi H , Diaz M , Qaiser N , Hussain MM . 2020 Soft actuators for soft robotic applications: a review. Adv. Intell. Syst. **2** , 2000128. (10.1002/aisy.202000128)

[3] Ilton M *et al* . 2018 The principles of cascading power limits in small, fast biological and engineered systems. Science **360** , eaao1082. (10.1126/science.aao1082) 29700237

[4] Baumgartner R *et al* . 2020 A lesson from plants: high-speed soft robotic actuators. Adv. Sci. **7** , 1903391. (10.1002/advs.201903391) PMC7055565 32154089

[5] Smith ML , Yanega GM , Ruina A . 2011 Elastic instability model of rapid beak closure in hummingbirds. J. Theor. Biol. **282** , 41–51. (10.1016/j.jtbi.2011.05.007) 21609721

[6] Hughes J , Culha U , Giardina F , Guenther F , Rosendo A , Iida F . 2016 Soft manipulators and grippers: a review. Front. Robot. AI **3** , 1–12. (10.3389/frobt.2016.00069)

[7] Shao H , Wei S , Jiang X , Holmes DP , Ghosh TK . 2018 Bioinspired electrically activated soft bistable actuators. Adv. Funct. Mater. **28** , 1–9. (10.1002/adfm.201802999)

[8] Bolmin O , Socha JJ , Alleyne M , Dunn AC , Fezzaa K , Wissa AA . 2021 Nonlinear elasticity and damping govern ultrafast dynamics in click beetles. Proc. Natl Acad. Sci. USA **118** , e2014569118. (10.1073/pnas.2014569118) 33468629 PMC7865152

[9] Chi Y , Li Y , Zhao Y , Hong Y , Tang Y , Yin J . 2022 Bistable and multistable actuators for soft robots: structures, materials, and functionalities. Adv. Mater. **34** , e2110384. (10.1002/adma.202110384) 35172026

[10] Wang Y *et al* . 2023 Insect-scale jumping robots enabled by a dynamic buckling cascade. Proc. Natl Acad. Sci. USA **120** , e2210651120. (10.1073/pnas.2210651120) 36689664 PMC9945960

[11] Holmes DP . 2019 Elasticity and stability of shape-shifting structures. Curr. Opin. Colloid Interface Sci. **40** , 118–137. (10.1016/j.cocis.2019.02.008)

[12] Daynes S , Weaver PM . 2013 Stiffness tailoring using prestress in adaptive composite structures. Compos. Struct. **106** , 282–287. (10.1016/j.compstruct.2013.05.059)

[13] Crooks W , Vukasin G , O’Sullivan M , Messner W , Rogers C . 2016 Fin Ray® effect inspired soft robotic gripper: from the robosoft grand challenge toward optimization. Front. Robot. AI **3** , 1–9. (10.3389/frobt.2016.00070)

[14] Groh RMJ , Avitabile D , Pirrera A . 2018 Generalised path-following for well-behaved nonlinear structures. Comput. Methods Appl. Mech. Eng. **331** , 394–426. (10.1016/j.cma.2017.12.001)

[15] Reis PM . 2015 A perspective on the revival of structural (in)stability with novel opportunities for function: from buckliphobia to buckliphilia. J. Appl. Mech. **82** , 11. (10.1115/1.4031456)

[16] Hu N , Burgueño R . 2015 Buckling-induced smart applications: recent advances and trends. Smart Mater. Struct. **24** , 063001. (10.1088/0964-1726/24/6/063001)

[17] Mendoza Nava H , Holderied MW , Pirrera A , Groh RMJ . 2024 Buckling-induced sound production in the aeroelastic tymbals of yponomeuta. Proc. Natl Acad. Sci. USA **121** , e2313549121. (10.1073/pnas.2313549121) 38315846 PMC10873622

[18] Jiang W , Dai F . 2021 An analysis for bi-stability characteristics of lateral buckled beams with energy dissipation features. Thin-Walled Struct. **169** , 108309. (10.1016/j.tws.2021.108309)

[19] Hu Y , Khezri M , Rasmussen KJR . 2022 Analytical solutions for buckling of space frames subjected to torsional loadings. Thin-Walled Struct. **173** , 108965. (10.1016/j.tws.2022.108965)

[20] Xiong Z , Chen L , Hao W , Yang P , Wang S , Wilkinson SL , Su Y *et al* . 2022 In-plane prestressed hair clip mechanism for the fastest untethered compliant fish robot. arXiv. (doi:arXiv:2207.08348)

[21] Dano ML , Hyer MW . 1998 Thermally-induced deformation behavior of unsymmetric laminates. Int. J. Solids Struct. **35** , 2101–2120. (10.1016/S0020-7683(97)00167-4)

[22] Seffen KA . 2007 ‘Morphing’ bistable orthotropic elliptical shallow shells. Proc. R. Soc. A **463** , 67–83. (10.1098/rspa.2006.1750)

[23] Pirrera A , Avitabile D , Weaver PM . 2012 On the thermally induced bistability of composite cylindrical shells for morphing structures. Int. J. Solids Struct. **49** , 685–700. (10.1016/j.ijsolstr.2011.11.011)

[24] Stratasys . 2018 FDM TPU 92A data sheet. See https://www.stratasys.com/siteassets/materials/materials-catalog/fdm-materials/tpu-92a/fdm-tpu-92a-3d-printing-material-data-sheet_a.pdf.

[25] Schafer BW , Li Z , Moen CD . 2010 Computational modeling of cold-formed steel. Thin-Walled Struct. **48** , 752–762. (10.1016/j.tws.2010.04.008)

[26] Shen J , Wadee MA , Sadowski AJ . 2017 Interactive buckling in long thin-walled rectangular hollow section struts. Int. J. Non Linear Mech. **89** , 43–58. (10.1016/j.ijnonlinmec.2016.11.007)

[27] Riks E . 1979 An incremental approach to the solution of snapping and buckling problems. Int. J. Solids Struct. **15** , 529–551. (10.1016/0020-7683(79)90081-7)

[28] Neville RM , Groh RMJ , Pirrera A , Schenk M . 2018 Shape control for experimental continuation. Phys. Rev. Lett. **120** , 254101. (10.1103/PhysRevLett.120.254101) 29979051

[29] Shen J , Groh RMJ , Schenk M , Pirrera A . 2021 Experimental path-following of equilibria using Newton’s method. Part I: theory, modelling, experiments. Int. J. Solids Struct. **210–211** , 203–223. (10.1016/j.ijsolstr.2020.11.037)

[30] Shen J , Garrad M , Zhang Q , Leao O , Pirrera A , Groh RMJ . 2023 Active reconfiguration of multistable metamaterials for linear locomotion. Phys. Rev. B **107** , 214103. (10.1103/PhysRevB.107.214103)

[31] Dong H , Chen CY , Qiu C , Yeow CH , Yu H . 2022 GSG: A granary-shaped soft gripper with mechanical sensing via snap-through structure. IEEE Robot. Autom. Lett. **7** , 9421–9428. (10.1109/LRA.2022.3187819)

[32] Chi Y , Hong Y , Zhao Y , Li Y , Yin J . 2022 Snapping for high-speed and high-efficient butterfly stroke-like soft swimmer. Sci. Adv. **8** , eadd3788. (10.1126/sciadv.add3788) 36399554 PMC9674291

[33] Divi S , Reynaga C , Azizi E , Bergbreiter S . 2023 Adapting small jumping robots to compliant environments. J. R. Soc. Interface **20** , 20220778. (10.1098/rsif.2022.0778) 36854379 PMC9974292

[34] Patek SN . 2023 Latch-mediated spring actuation (LaMSA): the power of integrated biomechanical systems. J. Exp. Biol. **226** , 226. (10.1242/jeb.245262) 37021687

[35] Rico-Guevara A , Rubega MA . 2017 Functional morphology of hummingbird bill tips: their function as tongue wringers. Zoology **123** , 1–10. (10.1016/j.zool.2017.06.001) 28760683

[36] Pishvar M , Harne RL . 2020 Foundations for soft, smart matter by active mechanical metamaterials. Adv. Sci. **7** , 1–17. (10.1002/advs.202001384) PMC7509744 32999844

[37] Shen J *et al* . 2024 Data from: A rapid-response soft end effector inspired by the hummingbird beak. Dryad Digital Repository. (10.5061/dryad.3bk3j9ktv) PMC11463234 39226926

[38] Shen J , Garrad M , Zhang Q , Wong VCH , Pirrera A , Groh RMJ . 2024 Supplementary material from: A rapid-response soft end effector inspired by the hummingbird beak. Figshare. (10.6084/m9.figshare.c.7402652) PMC11463234 39226926

